# Nutritional counseling in athletes: a systematic review

**DOI:** 10.3389/fnut.2023.1250567

**Published:** 2023-11-20

**Authors:** Simona Fiorini, Lenycia De Cassya Lopes Neri, Monica Guglielmetti, Elisa Pedrolini, Anna Tagliabue, Paula A. Quatromoni, Cinzia Ferraris

**Affiliations:** ^1^Human Nutrition and Eating Disorder Research Center, Department of Public Health, Experimental and Forensic Medicine, University of Pavia, Pavia, Italy; ^2^Laboratory of Food Education and Sport Nutrition, Department of Public Health, Experimental and Forensic Medicine, University of Pavia, Pavia, Italy; ^3^Department of Health Sciences, Sargent College of Health and Rehabilitation Sciences, Boston University, Boston, MA, United States

**Keywords:** nutritional counseling, athletes, nutrition knowledge, sport nutrition, nutritional strategies

## Abstract

Many studies report poor adherence to sports nutrition guidelines, but there is a lack of research on the effectiveness of nutrition education and behavior change interventions in athletes. Some studies among athletes demonstrate that nutrition education (NE), often wrongly confused with nutritional counseling (NC), alone is insufficient to result in behavior change. For this reason, a clear distinction between NC and NE is of paramount importance, both in terms of definition and application. NE is considered a formal process to improve a client’s knowledge about food and physical activity. NC is a supportive process delivered by a qualified professional who guides the client(s) to set priorities, establish goals, and create individualized action plans to facilitate behavior change. NC and NE can be delivered both to individuals and groups. To our knowledge, the efficacy of NC provided to athletes has not been comprehensively reviewed. The aim of this study was to investigate the current evidence on the use and efficacy of nutritional counseling within athletes. A systematic literature review was performed based on the Preferred Reporting Items for Systematic Reviews and Meta-Analyses method. The search was carried out in: PubMed, Scopus, Web of Science, Science Direct, Cochrane Library between November 2022 and February 2023. Inclusion criteria: recreational and elite athletes; all ages; all genders; NC strategies. The risk of bias was assessed using the RoB 2.0 Cochrane tool. The quality of evidence checking was tested with the Mixed Methods Appraisal Tool system. From 2,438 records identified, 10 studies were included in this review, with athletes representing different levels of competition and type of sports. The most commonly applied behavior change theory was Cognitive Behavioral Theory. NC was delivered mainly by nutrition experts. The duration of the intervention ranged from 3 weeks to 5 years. Regarding the quality of the studies, the majority of articles reached more than 3 stars and lack of adequate randomization was the domain contributing to high risk of bias. NC interventions induced positive changes in nutrition knowledge and dietary intake consequently supporting individual performance. There is evidence of a positive behavioral impact when applying NC to athletes, with positive effects of NC also in athletes with eating disorders. Additional studies of sufficient rigor (i.e., randomized controlled trials) are needed to demonstrate the benefits of NC in athletes.

**Systematic review registration:**

https://www.crd.york.ac.uk/prospero/, identifier CRD42022374502.

## 1 Introduction

Ensuring appropriate energy and nutrient intakes in athletes is critical in reaching and maintaining an optimal nutritional status, that supports peak performance and facilitates proper recovery after training and competition (1, 2). The nutritional requirements of athletes are influenced by numerous factors including gender, life stage, type of sport, training, phase of competition, environmental temperature, stress, high altitude exposure, physical injuries, phase of the menstrual cycle. For this reason, athletes’ dietary habits and diet composition should be customized according to their individual performance goals, preferences, and training phase (1). This work typically requires input from nutrition professionals known as registered dietitian nutritionists (RDNs) or accredited nutritionists, as terminology varies across the world.

Several authors report that many athletes do not meet their nutrition requirements (3) and do not have sufficient intakes of energy (4, 5), carbohydrates (6–8) and several micronutrients (5, 9). In contrast, some athletes seem to favor fat intake (8, 10, 11), which may be above the recommended levels (2), both considering the general sport requirements and the specific sport needs. The levels of inadequate intake are not surely known and can be influenced by the possible under-reporting (12–14) and are often based on the athletes’ limited nutrition knowledge (15–17). Moreover, athletes can adopt inappropriate and dysfunctional eating behaviors such as fasting, skipping meals, dieting, binge eating, vomiting, and use of laxatives and/or diet pills (18). Low sports nutrition knowledge and/or disordered eating behavior in the context of training and competing in sport can lead to the state of low energy availability (LEA) which is defined as the mismatch between energy intake and exercise energy expenditure (19). LEA in athletes may occur unintentionally (low awareness of the athletes’ nutritional needs), intentionally (attempts to optimize body composition for competition or to avoid weight gain during injury or illness), or compulsively (due to eating disorders [EDs] or disordered eating [DE] behavior) (20). One of the important health consequences of LEA is relative energy deficiency in sport (REDs), a syndrome characterized by a range of compromised physiological functions that negatively affect all body systems (i.e., metabolic rate, menstrual function, bone health, immunity, protein synthesis, cardiovascular health, gastrointestinal function, and more) (21, 22). LEA and REDs undermine athletic performance and put the athlete’s physical and mental health at risk (23), making these conditions prime targets for prevention and intervention activities (24).

For all these reasons, the development and application of valid intervention strategies is necessary to support athletes and protect their health. Bentley and colleagues (25) conducted a systematic review of the main sport nutrition interventions (i.e., nutrition education, nutritional counseling, individual and group workshops, consultations) applied to athletes, reporting that the up-to-date evidence-based literature is limited in its ability to identify the most effective strategy for use with this population. In spite of this, several studies reported that nutritional counseling (NC) could represent an important strategy to modify dietary habits and behaviors of athletes (26–35) making this a worthwhile area of investigation.

NC is a supportive process, characterized by a collaborative relationship between the counselor and the client(s) to establish food, nutrition and physical activity priorities, goals, and action plans (36). It is included in the Nutrition Care Process (NCP) model as a specific nutrition intervention generally delivered by RDNs (36). NC may apply a variety of models belonging to behavior change theories. The more widely used, validated theories are cognitive behavioral theory (CBT), social cognitive theory (SCT), transtheoretical model (TM), health belief model (HBF), systemic therapy (ST), and Mindfulness. These tools and strategies may be applied by themselves or in combination with other theories (i.e., CBT and TM together) based on the patients’ goals, objectives, and personal skills (36, 37). NC can be delivered both to individuals and groups.

It is important to identify NC as an intervention that is distinct from nutrition education (NE). NE is a formal process to instruct or help a patient in a skill or to impart knowledge to help clients voluntarily manage or modify food, nutrition, and physical activity choices and behavior to maintain or improve health (36). Designed to improve nutrition knowledge, its aim is to support sound food choices at the level of the community or within a specific target population (38, 39). In contrast, NC is a dynamic, two-way interaction that actively involves the client, using their existing nutrition knowledge as a starting point to define and support key behavioral changes. NC typically occurs in the context of an ongoing professional relationship where the nutrition counselor works privately with the client through a series of individualized sessions. The role of the sport nutrition counselor is to help athletes identify, adopt, and sustain a customized fueling strategy that maximizes training, performance, recovery, and holistic well-being while applying resources that facilitate nutritionally adequate, balanced eating patterns and address potential obstacles and barriers that predispose athletes to LEA and REDs. There is a role for both NE and NC when working with athletes, but the most appropriate strategy is one that is individually selected by the nutrition professional informed by their appraisal of the nutritional assessment, nutrition-related diagnosis, client needs, abilities, and life circumstances (36).

The role of the sport nutrition counselor is to offer advice to people interested in solving various current problems that the client(s) may face, which comprehend, for example, the ones derived from the preparatory work for performance in sports (i.e., energy balance according to training duration, type and intensity), contests and competitions participation, to promote the personal image, proposing a new perspective that can lead to overcoming the perceived or objective difficulties (e.g., fear of gaining weight, inadequate nutritional intake, management of injuries, etc.) and to optimize sport performances (40).

To the best of our knowledge, no review articles evaluated the application of NC in athletes to date. This paper aims to systematically review the current evidence on the use of NC in athletes and to identify the specific outcomes investigated to characterize its impact.

## 2 Materials and methods

This systematic review was performed based on the Preferred Reporting Items for Systematic Reviews and Meta-Analyses (PRISMA) method (41). The search was carried out in the following electronic databases: PubMed/MEDLINE, Scopus, Web of Science, ScienceDirect and Cochrane Library. The languages allowed were English and Italian according to the capability of comprehension of the authors. No limits were considered according to the date of publication. Randomized controlled trials, uncontrolled observational studies, case study, case reports and case series, opinion articles, conference abstracts, theses, and dissertations were included. The study protocol was previously submitted on the PROSPERO platform and has its registration number CRD42022374502.

### 2.1 Literature research strategy

An electronic search was conducted with subject index terms “nutritional counseling” OR “nutrition counseling” OR “nutritional and eating education” OR “nutritional program” combined with the term “athletes” OR “sports” OR “performance” OR “athletic performance” OR “recreational athletes” OR “elite athletes.” Google Scholar was used to search gray literature and some references found in review articles were included manually. The populations of interest were recreational and elite athletes. We did not specify comparison conditions in our search because this was not included in the aim of the study which was simply to evaluate the use of NC, not necessarily compared to other strategies. The search strategy is illustrated in Table 1. Detailed criteria for study inclusion and exclusion are listed in Table 2.

**Table 1:** Search strategy for different databases.

Data base	Search strategy	Number of articles
PubMed	(“Nutritional counseling” [All Fields] OR “Nutritional counselling” [All Fields] OR “Nutrition Program” [All Fields] OR “Nutritional education” [All Fields] OR “EATING education” [All Fields]) AND (“athletes” [MeSH Terms] OR “sports” [MeSH Terms] OR “athletic performance” [MeSH Terms] OR “recreational athletes” [All Fields] OR “elite athletes” [All Fields])	98
Scopus	TITLE-ABS-KEY ((“Nutritional counselling” OR “Nutritional counselling” OR “Nutrition Program” OR “Nutritional education” OR “EATING education”) AND (“athletes” OR “sports” OR “athletic performance” OR “recreational athletes” OR “elite athletes”))	230
Web of Science	(“Nutritional counselling” OR “Nutritional counselling” OR “Nutrition Program” OR “Nutritional education” OR “EATING education”) AND (“athletes” OR “sports” OR “athletic performance” OR “recreational athletes” OR “elite athletes”)	79
Science Direct	(“Nutritional counselling” OR “Nutritional counselling” OR “Nutrition Program” OR “Nutritional education” OR “EATING education”) AND (“athlete$” OR “sports”)	1948
Cochrane reviews	(“Nutritional counselling” OR “Nutritional counselling” OR “Nutrition Program” OR “Nutritional education” OR “EATING education”) AND (“athletes” OR “sports” OR “athletic performance” OR “recreational athletes” OR “elite athletes”) in Title Abstract Keyword	82

**Table 2:** PICOS criteria of inclusion and exclusion.

PICOS criteria	Inclusion criteria	Exclusion criteria
Population	Recreational and elite athletes, all ages, all genders	General population
Intervention	Nutritional Counseling strategies	No nutritional intervention
Comparison	Not applicable	Not applicable
Outcomes	Adherence, compliance rates, nutrition knowledge, eating disorders, REDs, athlete triad, injuries, performance, body image, body dissatisfaction, low energy availability, osteopenia, amenorrhea, anemia and others	Not applicable
Types of studies included	Randomized controlled trials; uncontrolled intervention studies; case study, case reports and case series, opinion articles, conference abstracts, theses, and dissertations	Full text not available; without the outcomes of interest; reviews, guidelines, letters, editorials, comments, news, *in vitro* or animal studies
Research question	What evidence do we have to deliver nutritional counseling to athletes, and to impact what specific outcomes?	

### 2.2 Study selection

The research and study selection was carried out by two authors (EP and LCLN) independently using the Rayyan software (42), following two steps. First, authors read the titles and abstracts; next, they evaluated the full articles selected in the previous stage, and included other relevant studies found in the reference lists of the selected articles. The decision to include an article was based on the PICOS strategy (Population (P): athletes; Intervention (I): nutritional counseling; Control (C): placebo, Outcome (O): adherence/compliance rates, nutrition knowledge, eating disorders, REDs, athlete triad, injuries, performance, body image, body dissatisfaction, low energy availability, osteopenia, amenorrhea, anemia and others; Study type (S): Randomized controlled trials, uncontrolled observational studies, case study, case reports and case series, opinion articles, conference abstracts, theses, and dissertations). When disagreement was found, a third author (SF) reviewed the full text articles to decide about inclusion.

Adherence, compliance rates, nutrition knowledge, eating disorders, REDs-S, athlete triad, injuries, performance, body image, body dissatisfaction, low energy availability, osteopenia, amenorrhea, anemia.

The risk of bias was also assessed by two authors, independently and blinded (EP and LCLN) using the RoB 2.0 Cochrane tool (43), checking 5 domains: (1) Randomization process, (2) deviations from intended interventions, (3) missing outcome data, (4) measurement of the outcome and (5) selection of the reported result. When disagreement was found, a third author (SF) decided. This tool was applied only to the clinical trials because of the adequacy of the instrument in this specific study design and the lack of control groups in the other reports.

The quality of evidence checking was tested for all articles with the Mixed Methods Appraisal Tool system (MMAT) (version 2018) (44) by two authors, independently and blinded (EP and LCLN). When disagreement was found, a third author (SF) decided. Data tables were constructed based on the articles’ details.

## 3 Results

**Figure 1:**
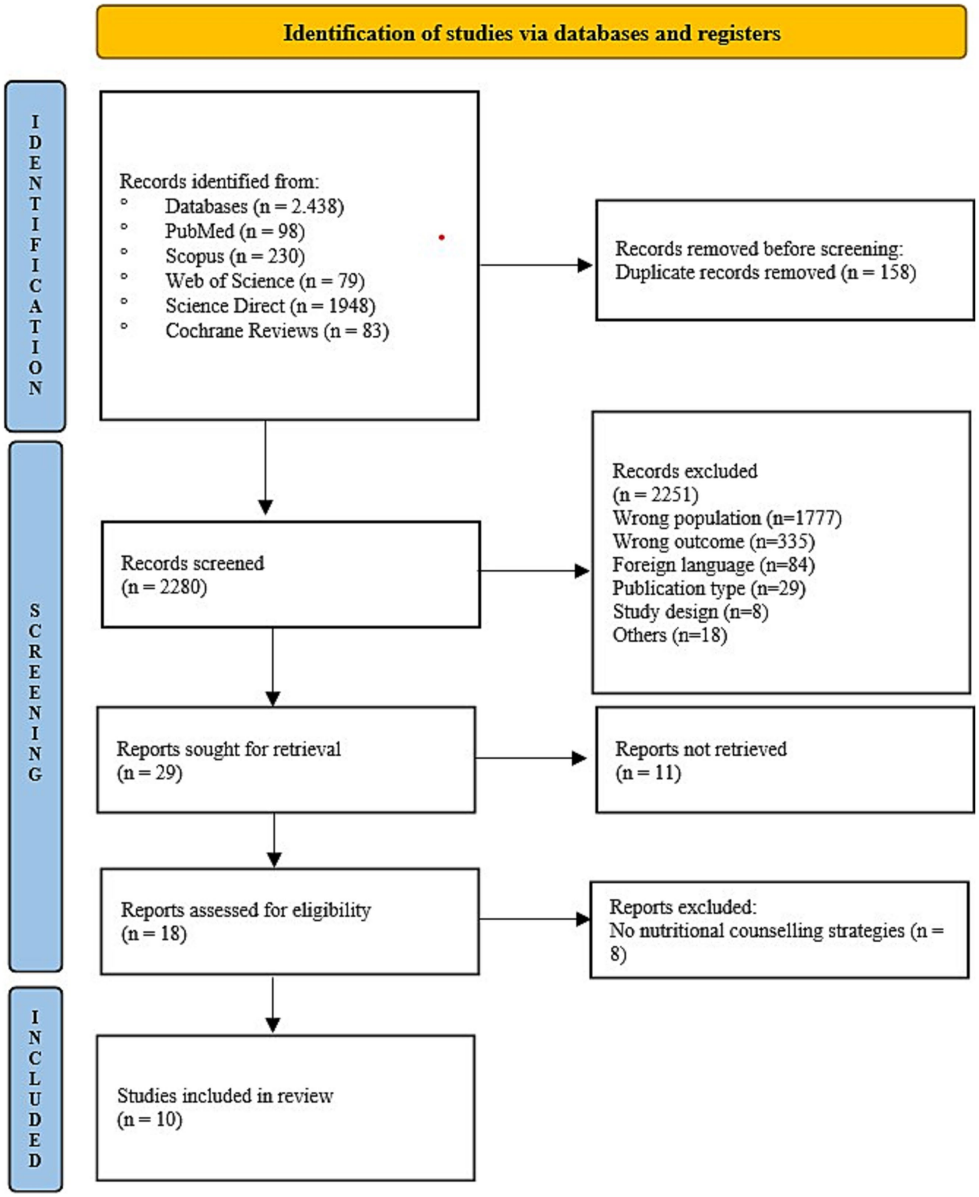
Preferred Reporting Items for Systematic Reviews and Meta-Analyses (PRISMA) flow chart.

A total of 2,438 records were identified through database searches. After removal of duplicates, 2,280 articles remained. After first screening by title and abstract, 29 records were sought for retrieval. The indications for excluding 2,251 articles are shown in Figure 1. Eighteen articles were retrieved. Upon reading, eight articles were excluded because they did not use nutritional counseling strategies. Ten studies were included in this review (Figure 1).

### 3.1 Study characteristics

The selected studies are summarized in Table 3. All were published between 2004 and 2022. Four studies were conducted in the United States (26, 33–35), two in Norway (27, 28), one in Poland (29), one in Algeria (32), one in Canada (31), and one in Finland (30).

**Table 3:** Details of included articles: population characteristics, type of intervention, results and quality of evidence (Mixed Methods Appraisal Tool system [MMAT]).

Study, year, country	Design of study	Population characteristics	Nutritional counseling intervention		Outcomes	Results	Quality (MMAT)
			Strategy and duration	Intervention characteristics			
**Randomized clinical trials studies**							
Abood et al., 2004, United States (26)	Randomized cross over study	*N* = 30 F, equally distributed in soccer team players (intervention group) swim team players (control group) Age, y (Mean ± SD): 19.6 ± 1.0 and 19.4 ± 1.2, respectively BMI, kg/m^2^ (Mean ± SD): 21.3 ± 2.4 and 21.9 ± 2.6, respectively, type of sport practiced: not specified	SCT - group counseling: Social support Problem solving Skill training/demonstration Duration: 8 sessions on weekly basis	1-h self-efficacy educational sessions Professionals: not mentioned	Collected pre and post nutrition knowledge and self-efficacy questionnaires and a 3-day diet record	Intervention group improved: Nutrition knowledge Self-efficacy (*p* < 0.05) Overall number of positive dietary changes (*p* < 0.03)	****
Garthe et al., 2011, Norway (27)	Randomized cross over study	*N* = 21 elite 4F/17M athletes divided in 2 groups: NCG n = 12: 2F/10M, Age: 18.5 ± 1.7 years. ALG n = 9: 2F/7M, Age:19.6 ± 2.7 years. BMI, kg/m^2^ (Mean ± SD): n.d. Type of sport (%): NCG: Ice hockey (23.8), soccer (9.5), skating (9.5), volleyball (4.8), taekwondo (4.8) and rowing (4.8) ALG: ice hockey (19), soccer (9.5), skating (9.5), kayaking (4.8)	Theory-based - Group counseling Self-monitoring Duration: 8–12 week (targeted on BM gain objective)	NCG athletes received NC 1/week for 8–12 week on basic nutrition, sports physiology and possible adjustments in the dietary plan or weight regimen ALG athletes were instructed to eat *ad libitum* 4 strength training sessions/week were included After the intervention period, all athletes received 1 NC session and 1 exercise counseling session to stabilize their new BM and body composition Professionals: 2 experienced nutritionists, one clinical dietician and one exercise physiologist specialized in sports nutrition	BW DXA Training (h per week) Diet (4-day weighed food record)	In NCG: ↑ Energy intake compared to baseline and ALG ↑ BW ↑ LBM	*****
Garthe et al., 2013, Norway (28)	Randomized cross over study	*N* = 39 elite 3F/36M athletes divided in 2 groups: NCG n = 21, 2F/19M, Age: 19.2 ± 2.9 years ALG n = 18, 1F/17M, Age: 19.7 ± 2.7 years BMI, kg/m^2^ (Mean ± SD): n.d. Type of sport (%): ice hockey (66.7), skating (12.8), rowing (2.6), kayaking (2.6), volleyball (2.6), taekwondo (2.6)	Theory-based - Group counseling and Individual diet Self-monitoring Duration: 8–12 week (targeted on BM gain objective)	NCG athletes received NC one a week for 8–12 week on basic nutrition, sports physiology and possible adjustments in the dietary plan or weight regimen + personalized diet; ALG athletes were instructed to eat *ad libitum* 4 strength training sessions/w were included Professionals: 2 experienced nutritionists, one clinical dietician and one exercise physiologist specialized in sports nutrition	BW DXA Training (h per week) Diet (4-day weighed food record) Strength (1RM, 40 m sprint and CMJ)	NCG ↑ energy intake and BW FM increased more in NCG than ALG	*****
Grabia et al., 2022, Poland (29)	Randomized cross over study	*N* = 46 adolescents (*n* = 28 NC group; *n* = 18 non-education group) Gender, not mentioned Age, years (Mean ± SD): n.d. Min-Max: 14–16 years BMI, kg/m^2^ (Mean ± SD): n.d. Type of sport: soccer	Theory-based - Group education and counseling Problem solving Motivational interviewing Duration: 17 weeks	NC group: 7-part program consisting of sections on motivation, nutritional recommendations for young athletes, peri-exercise nutrition and hydration, supplementation, common nutritional mistakes and improper eating habits Professionals: Not mentioned	Prohealthy-Diet-Index-10 (pHDI-10) Non-Healthy-Diet-Index-14 (nHDI-14) Diet: dietary interviews from 3 days BW Blood sample analysis	In NC group: ↓ Saccharose intake ↑ Digestible carbohydrates, fiber, fluid above 2 L/day Reported by athletes better peri-workout hydration, well-being, faster regeneration	***
Heikkilä et al., 2019, Finland (30)	Randomized controlled trial	*N* = 79 endurance athletes (EDU group n = 37 18F/19M, EDU + APP n = 42 17F/25M) Age, y (Mean ± SD): 18 ± 14 BMI, kg/m^2^: n.d. Type of sport: cross-country skiing endurance running/race-walking biathlon, orienteering, triathlon	Self-determination theory - Group education and counseling Skill training/demonstration Motivational interviewing Self-monitoring Goal setting Active learning Duration: 3 sessions +17-weeks follow-up	3 sessions of nutritional education (90 min) Professional: Nutritionist	Nutrition knowledge evaluation through a validated questionnaire Diet: 3-day food diary	Both groups: ↑ nutrition knowledge during intervention Use of the mobile app not improve the results further	****
Laramée et al., 2017, Canada (31)	Cluster randomized controlled trial	*N* = 70 adolescent F athletes (intervention group = 37; control group = 33) Age, y (Mean ± SD): 14.1 ± 1.5 and 13.1 ± 1.2, respectively, BMI, kg/m^2^ (Mean ± SD): n.d. categories (%) Intervention group: underweight (8), normal weight (73), overweight (19) Control group: underweight (12), normal weight (73), overweight (12), obese (3) Type of sport (%) Intervention group: synchronized swimming (87), dance (14) Control group: synchronized gymnastic (42), cheerleading (58)	TPB - Group Counseling Persuasive communication Active learning Observational modeling Duration: 3 weeks +2–3 month follow-up	Both groups had nutrition education during 3 weekly 60-min sessions. In the intervention group a 3 sessions 60-min TPB was added, targeting the specific determinant of intention to use restrictive dietary behaviors for losing weight Professionals: Registered dietitian with formal training in the management of disordered eating and experience in working with adolescents	BW Nutrition knowledge evaluation through 37 multiple choice and true/false questions Psychosocial determinants: Intention, attitude, subjective norm and perceived behavioral control.	Intervention group: ↓ intention of using restrictive dietary behaviors for losing weight over time Both groups: ↑ nutrition knowledge score	*****
**Longitudinal and observational studies**							
Sahnoune et al., 2020, Algeria (32)	Longitudinal study, pre and post design (no control group)	*N* = 80 - 39F/41M Age, year (Mean ± SD): 15 ± 1.0 BMI, kg/m^2^ (Mean ± SD): 20.7 ± 2.8 Type of sport (%): hand-ball (37.5); athletics (33.7); swimming (11.2); judo (8.8); basketball (8.8)	Theory-based - Individual counseling + group intervention Skill training/demonstration Duration: 6 months	6 Nutritional group intervention + face-to-face individual counseling after each workshop Professionals: Nutritionist	BW Adherence mediterranean diet: KIDMED index Diet: 24 h recall	Athletes increased: Mediterranean Diet adherence Energy, fiber, complex carbohydrates and protein intake	***
Stranberg et al., 2020, United States (33)	Longitudinal study, pre-post design (no control group)	*N* = 15 F athletes with eating disorder diagnoses: OSFED (9), AN (5), BN (1) Age, y (Mean ± SD): 20.9 ± 5.2 BMI, kg/m^2^ (Mean ± SD): 21.4 ± 2.6 Type of sport (%): distance running (40), ball sports (27), ice hockey (13), body building (13), triathlon (7)	CBT, DBT, − Group and individual counseling Goal setting Relapse prevention Self-monitoring Motivational interviewing CBT and DBT strategies Duration: Average duration was treatment was 8 weeks	Minimum of 18 treatment sessions: 6-week series of group lessons addressing mental health and nutrition for ED recovery Weekly individual sessions with the dietitian and separately with the mental health provider Professionals: Multidisciplinary team of mental health clinicians, RDN, sport psychologists, and exercise science professionals with dual training in eating disorders and sport	Anthropometric measures Eating disorder behaviors, and compliance with meal plan and exercise prescriptions EDE-Q Satter Eating Competence Inventory (ecSI 2.0) FAST evaluation of eating disorder risk	1.5% increase in body weight on average Decrease in EDE-Q global score and all subscale scores Increase in eating competence score and all subscores, with 1/3 of patients achieving eating competence by discharge Decrease in FAST score, with 2/3 of athletes in healthy range, 1/3 in subclinical eating disorder range, none in the clinical ED range by discharge Qualitative evidence of impact of treatment that included NC interventions	****
Quatromoni, 2008, United States (34)	Case series	*N* = 68 (n = 25 in the first year) 49F/19M Age, y (Mean ± SD): n.d. BMI, kg/m2 (Mean ± SD): from 23.7 to 24.5 on average for all female subgroups, n.d. for men Type of sport: 32% lean sports (swimming, track and cross country), plus crew, basketball, ice hockey, field hockey, lacrosse, golf, soccer, softball, and tennis	CBT, Individual counseling Problem solving Motivational interviewing Self-monitoring Journaling Goal setting Cognitive restructuring Relapse prevention Assessment of readiness to change Individualized meal plans Eating pattern advice Duration: Up to 2 years (depends on specific case)	Professionals: Registered dietitian with expertise in sports nutrition	FAST evaluation of eating disorder risk Self-reported height and weight for calculation of BMI Assessment and monitoring of nutritional adequacy	>50% of women athletes had FAST scores indicative of subclinical or clinical disordered eating across a range of sports and body sizes	***
Quatromoni, 2017, United States (35)	Dual case study	*N* = 2 athletes with eating disorder diagnoses: *n* = 1 F with AN *n* = 1 M with EDNOS Age, y (Mean ± SD): n.d. BMI, kg/m^2^ (T0): 16.4 (F); 20.8 (M) Type of sport: track and field	CBT and DBT- Individual counseling and education Motivational interviewing Self-monitoring CBT and DBT strategies Duration: 5 years	Female athlete Outpatient nutritional counseling and education + weekly sport psychology sessions provided CBT and DBT Male athlete Outpatient nutritional counseling and education (CBT + DBT) + motivational interviewing Professionals: Multi-disciplinary sports medicine team, including a registered dietitian, sport psychologist, sports medicine and athletic trainers	Demographics ED onset Clinical and behavioral presentation FAST evaluation of eating disorder risk (female athlete only) Diet: 24-h dietary recalls, food records, and a diet history interview Anthropometric measures (BW) and body composition assessment (male athlete only) Psycho-social presentation Outpatient ED treatment	Both athletes: Restored weight, this allowed them to achieve sport/performance goals Achieved recovery from ED Once recovered, publicly acknowledged their ED and how it negatively impacted their sport Maintained recovery, 7 years later Still competing at an elite level	***

BW, body weight; LBM, lean body mass; FM, fat mass; F, female; M, male; RDN, registered dietitian nutritionist; FAST, Female Athlete Screening Tool; BMI, Body Mass Index; CBT, Cognitive Behavioral Therapy; SCT, Social Cognitive Theory; NCG, Nutritional Counseling Group; ALG, Ad Libitum Group; TPB, theory of planned behavior; DXA, dual-energy X-ray absorptiometry; 1RM, one repetition maximum; CMJ, countermovement jump; ED, eating disorder; FAST, Female Athlete Screening Tool; EDE-Q, Eating Disorder Examination Questionnaire; OSFED, Other Specified Feeding or Eating Disorder; DBT, dialectical behavioral therapy; EDNOS, eating disorder not otherwise specified; EDU, education session group.

A variety of study designs are represented in this sample. There were four randomized cross-over studies (26–29), one randomized controlled trial (30), one cluster randomized controlled trial (31), two longitudinal studies (32, 33), one case series (34), and one dual case study (35). No studies from grey literature were included.

### 3.2 Participant demographics and characteristics

The studies reported on a combined total of 450 participants, mainly females (60.9%). One article did not report the athletes’ gender. Sample sizes ranged from two (35) to 80 participants (32). Three studies (34–36) involved athletes with eating disorders.

Participants were adolescent athletes, college students, elite athletes (qualified for national teams or members of a recruiting squad), and national or international level athletes. The sports represented in the study samples included: soccer, swimming, track and cross country, crew, basketball, ice hockey, field hockey, lacrosse, tennis, golf, rowing, volleyball, softball, taekwondo, skating, kayaking, synchronized swimming, gymnastics, dance, cheerleading, cross-country skiing, endurance running/race-walking, biathlon, orienteering, triathlon, bodybuilding, athletics, judo.

### 3.3 Study interventions (exposures)

#### 3.3.1 Nutritional counseling types, strategies and duration

Six studies (26–31) delivered group counseling, one study employed both group and individual counseling (33), and three studies used only individual counseling (32, 34, 35). The type of NC delivered was specified only in 6 articles, with three of them using multiple strategies (33–35). The most commonly used was CBT (33–35), combined with Dialectical Behavioral Therapy in two studies (33, 35). Abood et al. delivered nutritional counseling based on social cognitive theory using self-efficacy educational sessions (26). Laramée et al. focused on behavior change using the theory of planned behaviour targeting the specific determinant of intention to use restrictive dietary behaviors for losing weight (31). Grabia et al. dedicated one session of the program to motivation (29).

More specifically, 15 different strategies were applied across the interventions, described in Table 3. The most frequent strategies used were ‘motivational interviewing’ (29, 30, 33–35) and “self-monitoring” (27, 28, 30, 33–35), followed by “problem-solving” (26, 29, 34), “goal setting” (30, 33, 34) and “skills training” (26, 30, 32). Quatromoni used a combination of ten different strategies across a sample of athletes (34).

Eight studies reported the topics of intervention, such as caloric intake and energy expenditure, carbohydrates, fats and proteins, fluids, calcium, iron, and zinc (*n* = 6), dietary challenges such as eating on the road (*n* = 4), restrictive dietary behaviors and disordered eating (*n* = 4), supplement use (*n* = 2), Mediterranean Diet pyramid, typical athlete meal, healthy food recipes (*n* = 1), nutritional recommendations for young athletes (*n* = 1), peri-exercise nutrition and hydration (*n* = 2), sports physiology and possible adjustments in the dietary plan for weight regimen (*n* = 2), fueling for sport and for life, eating competence, body esteem, recovery skills, resilience (*n* = 1). Some topics were common in the different studies, so some themes are repeated in the count.

The duration of the intervention and/or follow-up was highly varied. The most brief intervention lasted 3 weeks (31) and the longest had a duration of 5 years (35). Only three studies reported the duration of each session. In the studies by Abood et al. (26) and Laramée et al. (31), the sessions were 60 min long, and in the study by Heikkilä et al. (30), workshops duration was 90 min.

In the reviewed studies, nutritional counseling was delivered by a RDN (31, 34), a multidisciplinary team in which an RDN was involved (33, 35), a nutritionist (30, 32), or two experienced nutritionists (one clinical dietitian and one exercise physiologist specialized in sports nutrition) (27, 28). Two studies did not report the qualifications or discipline of the facilitator (26, 29).

### 3.4 Study results (outcomes)

The possible outcomes were categorized into Questionnaires results and Nutritional Counseling Efficacy, which is more specifically divided in (1) nutrition knowledge, (2) dietary intake and (3) remission from eating disorders.

#### 3.4.1 Questionnaires

Three studies (26, 30, 31) administered nutrition knowledge questionnaires to evaluate differences between pre- and post-intervention scores. Three studies (33–35) administered the Female Athlete Screening Tool (FAST) that screens for athlete-specific eating disorder risk (45). Stranberg et al. (33) also used the Eating Disorder Examination Questionnaire (EDE-Q) that evaluates the frequency and severity of eating disorder behavior (46) and the Satter Eating Competence Inventory (ecSI 2.0) that assesses eating competence, which includes rebuilding a healthy relationship with food, developing skills for meal planning and reliably feeding oneself and applying informed intuitive eating (47). Abood et al. (26) used a self-efficacy questionnaire and Laramée et al. (31) used a questionnaire of psychosocial determinants of intention to use restrictive dietary behaviors for losing weight. Anthropometric data were collected in all 10 studies. Body composition was assessed in three studies (27, 28, 34).

Seven studies assessed dietary intake and nutritional adequacy, using either a 3-day food record (26, 30, 31), a 4-day weighed food record (27, 28), a 24-h dietary recall (32), a combination of tools for dietary assessment and monitoring (34, 35), or a questionnaire that informed the calculation of the ProHealthy-Diet-Index-10 (pHDI-10) and the Non-Healthy-Diet-Index-14 (nHDI-14) (29). An individualized meal plan was part of the intervention in five studies (27, 28, 33–35).

#### 3.4.2 Nutritional counseling efficacy

##### 3.4.2.1 Nutrition knowledge

Three studies (26, 30, 31) showed an improvement in nutrition knowledge in athletes to whom nutritional counseling was delivered. Laramée et al. (31) highlighted an improvement in nutrition knowledge in both control and intervention groups, but only in the intervention group was there a decrease in the intention of using restrictive dietary behaviors for losing weight.

##### 3.4.2.2 Dietary intake

Results of three studies (26, 29, 32) showed some measurable changes in dietary intake. Athletes increased adherence to the Mediterranean Diet and energy, fiber, carbohydrate and protein intake (32), increased fluid intake above 2 L/day, and decreased sugar intake (29). In the study by Abood et al. (26), the nutritional counseling group significantly improved self-efficacy (*p* < 0.05) and achieved an overall higher number of positive dietary changes (*p* < 0.03) compared to those in the control group who did not receive any treatment. Two studies (27, 28) showed an increased energy intake and consequently, increased body weight, in the Nutritional Counseling Group (NCG) compared to baseline and compared to the *Ad Libitum* Group (ALG) that received no nutritional counseling intervention. In both studies, athletes in the NCG increased fat mass and lean body mass to a greater extent than athletes in the ALG.

##### 3.4.2.3 Remission from eating disorder

From a longitudinal observational study of 15 college female athletes who underwent nutritional counseling in the setting of an intensive outpatient program for the treatment of eating disorders in sport, Stranberg et al. (33) reported a decrease in the FAST score where two thirds of athletes scored in the healthy range and only one-third scored in the subclinical eating disorder range at discharge. This was in contrast to only 32% in the healthy range, 26% subclinical, and 42% screening positive for a clinical ED on admission. Further evidence of remission from the eating disorder and achieving normalized eating patterns was provided by this study where a measurable decrease in EDE-Q scores and an increase in eating competence scores was shown as a consequence of the intervention. While only 10% of athletes were competent eaters on admission, 33% were on discharge. More than half of admissions resulted in weight gain (58%). The dual case study that applied NC to athletes with eating disorders in the outpatient setting (35) reported the achievement of weight restoration (evidence of nutritional adequacy) and recovery from the eating disorder, with recovery maintained 7 years later after treatment by a multidisciplinary team that included a registered dietitian.

### 3.5 Study quality

The quality of evidence checking was tested using the Mixed Methods Appraisal Tool system (MMAT) (version 2018) (44). The results are reported in Table 3. Four studies reached three stars (29, 32, 34, 36), three studies (26, 30, 33) were evaluated with four stars, and three studies earned the maximum five stars (27, 28, 31).

**Figure 2:**
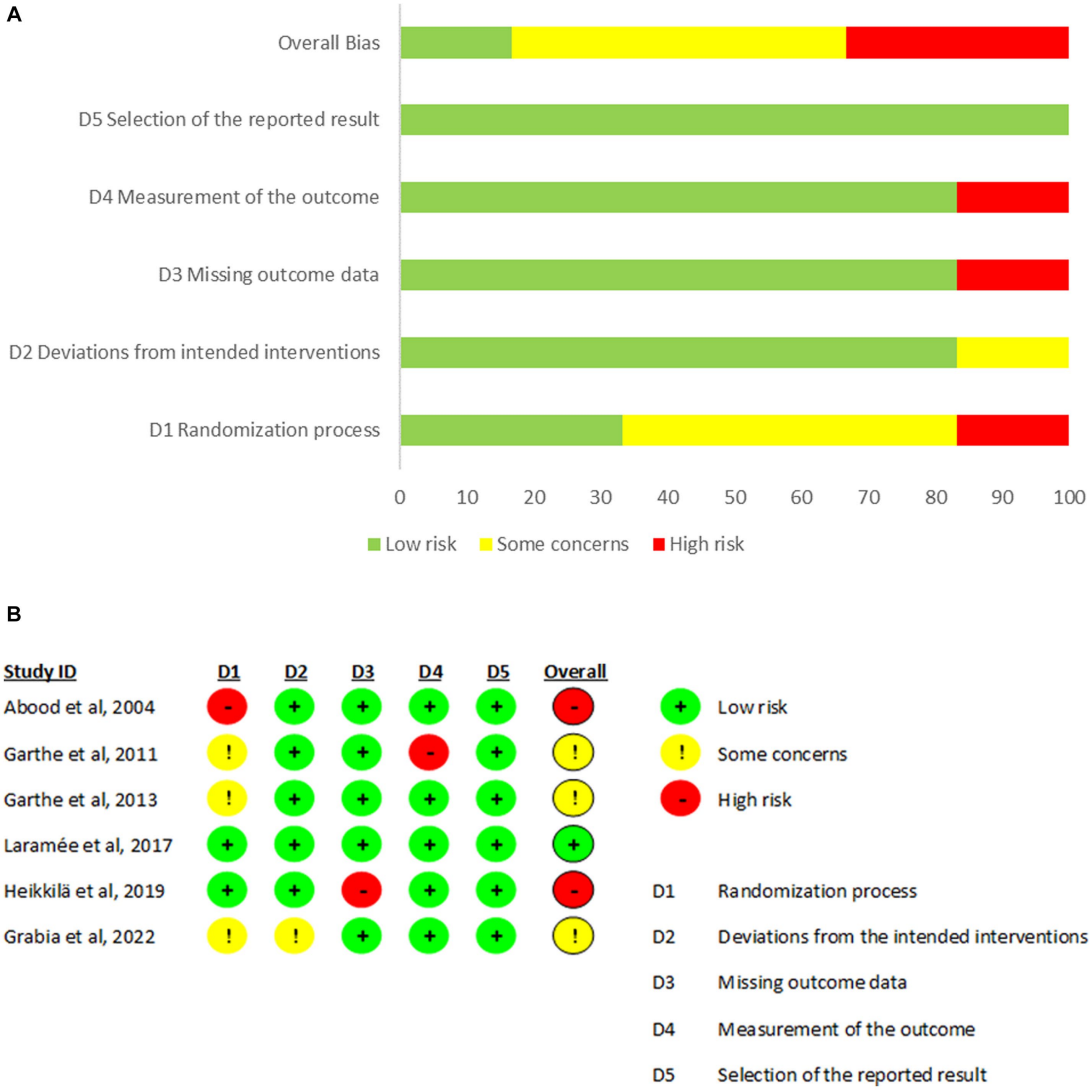
Results of risk of bias analysis. **(A)** Percentage of risk of bias of each domain in all included studies. **(B)** Description of each domain of bias according to studies included (43).

The risk of bias was assessed using the RoB 2.0 Cochrane tool (Figure 2) (43) according to the study procedures for six studies (26–31). Overall results showed one study at low risk of bias (31), three studies with some concern (27–29), and two studies at high risk of bias (26, 30). The randomization process was the domain at higher risk of bias. For overall studies, the domain ‘selection of the reported results’ was at low risk of bias. Domains 2, 3, and 4 (deviations from the intended intervention, missing outcome data, measurements of the outcome) obtained a low-to-moderate risk of bias score for all studies except for Garthe et al. (27) and Heikkila et al. (30) for domains 4 and 3, respectively, where the risk was high.

## 4 Discussion

To our knowledge, this is the first systematic review that evaluates the delivery of nutritional counseling to athletes and summarizes its potential effectiveness. Each of the ten studies reviewed reported some beneficial changes in the diets or eating behaviors of athletes as a consequence of nutritional counseling interventions. The main results showed that NC interventions induced positive changes in nutrition knowledge, dietary intake (quality and/or adequacy) consequently affecting athletic performance, and recovery from eating disorders. While more research is needed on this topic, these initial observations support the inclusion of nutrition professionals in sport environments to make NC interventions accessible and impactful.

Across the ten studies included in this review, there was a substantial amount of heterogeneity in key areas that affect the interpretation of this evidence. Authors used a variety of NC strategies grounded in different behavioral change theories. The duration of the counseling interactions ranged from weeks to years, and in some instances was determined by individualized needs such as treatment for REDs or an eating disorder. Most of the intervention plans delivered group counseling, some combined group and individual counseling strategies, and others only involved individual counseling. Finally, population characteristics varied widely. Samples more often included females, but some studies did include males and they involved athletes of different ages and levels of competition, and the types of sports practiced were quite varied. While this gives some sense of comprehensive diversity and inclusion, it is somewhat challenging to draw definitive conclusions.

In spite of the heterogeneity in this literature, all of the studies reported outcomes that can be interpreted as desirable in terms of athlete nutritional status and well-being. In particular, the case report by Quatromoni et al. (35) described in detail the outpatient treatment of two collegiate track athletes with diagnosed eating disorders. During the NC sessions, the dietitian used strategies based on CBT, DBT, and MI. Both athletes achieved weight restoration and recovered from anorexia and ED reaching sport performance goals, and maintaining recovery years out from treatment. Stranberg et al. (33) reported similar results in a sample of 15 female athletes with eating disorder diagnoses. This latter paper (33) is the first one that documents the low level of eating competence in athletes treated for EDs, and shows how NC interventions that target personal feeding skills and eating behaviors are relevant, effective, and aligned with ED recovery. Similarly, the study conducted by Laramée et al. (31) demonstrated a decrease in restrictive eating behavior in the group of athletes exposed to NC.

Other studies documented important positive modifications in terms of nutrition knowledge and dietary intake among athletes provided NC from nutrition professionals (27). It is well established that a balanced and adequate diet plays an important role in maintaining health, allowing athletes to perform at a high level, and recover from the stress of training and competition more efficiently (1, 48). To apply the principles of sports nutrition, basic knowledge and understanding of nutrition are necessary; however, knowledge does not necessarily translate to behavior and it may not be sufficient to allow athletes to thrive and reach their full potential. The literature on NE is growing (49–51), intending to support optimal eating patterns within the community or a specific target population such as athletes. The addition of NC on top of NE appears essential. In fact, recent research has shown that NE programs may be less effective at inducing positive dietary changes (17). Otherwise NC combines information with strategies to achieve a behavior change based on individual characteristics, beliefs, and goals, setting it apart as its own worthy intervention approach.

Moreover, the sports environment often influences athletes’ eating behaviors in ways that undermine nutritional adequacy and sabotage well-being and performance (obtaining a sport-specific body type, academic stress, team culture, coach and family expectations, and social perceptions or norms) (52, 53). Some types of sport have been shown to be more related to the development of disordered eating and ED, specifically track, cross-country, cycling, swimming, gymnastics, dance, figure skating, and judo (18), whereas other literature (54, 55) suggests that ED in sport are more widespread, do not discriminate by sport, gender or body type, and after quite under-reported, under-diagnosed and under-treated. Pressure from athletes’ teammates or training groups may also play an integral role in the development and maintenance of athletes’ eating/exercise psychopathology with both direct comments about weight and body shape noted as contributing factors, and indirect influences from peer modeling and social media content playing a role (54, 55).

For all of these reasons, an effective approach is needed to support athletes’ nutritional well-being given its important interrelationship with physical health, mental health, and sports performance. Evidenced by the studies included in this review, nutritional counseling could represent a functional strategy in this pursuit. From our review it can be seen that CBT has been the most widely used NC theory. Furthermore, the most used strategies were motivational interviewing and self-monitoring, although the importance of a combination of different strategies has emerged. There were some limitations in this systematic review. Aside from the heterogeneity of the athlete samples and study designs used, there was a lack of specification of the type of NC techniques used in some studies. These factors prevent us from addressing a more detailed hypothesis on whether (and which) specific NC theories or strategies could be more suitable or impactful, in general or for a specific group of athletes. Moreover, nutritional counseling was not a keyword recognized in MESH terminology. This may contribute to a possible under-estimation of the actual number of studies to be evaluated if some were missed for this reason. Some reports constitute observations from clinical practice and, while more detailed in nature and certainly important to guide practice when research on a topic is limited, these reports lack the rigor of randomized controlled trials designed specifically to test hypotheses about treatment outcomes from interventions like NC. The four studies that were not clinical trials were of moderate quality, and they are naturally subject to more potential risk of bias than the RCTs. Although each of the six RCTs had a low risk of bias in the majority of the domains considered, only one trial had an overall low risk of bias.

This review is also supported by some strengths. First, scientific literature on the topic of NC in athletes is scarce, so this article provides the opportunity for critical thinking on this topic and a roadmap for future research. Second, this line of research strictly differentiates nutritional counseling from food education interventions, demonstrating the added value and the unique role that nutrition professionals bring to the sports environment. Actually, RDNs who specialize in sports and human performance nutrition (i.e., sports RDNs) are the preferred providers of NC interventions because of their extensive and varied training experiences that include clinical nutrition and medical nutrition therapy (MNT), education and behavioral counseling, food service and culinary nutrition, exercise physiology, and evidence-based nutrition guidance for physical performance (56). Advanced training that allows the RDN to engage in screening, assessment, treatment, and prevention of REDs and eating disorders in sport is evidenced in this review. Considering the small yet emerging literature on this topic, almost half of the studies reviewed had a minimum quality rating of four stars using the MMAT method.

## 5 Conclusion

Nutritional counseling induces positive, measurable behavioral effects in athletes, improving nutrition knowledge, fostering the adoption of adequate eating patterns, and supporting recovery from REDs and ED in sport. There is, however, a lack of homogeneous research, in terms of design, population and methods, involving nutritional counseling provided to athletes which makes it difficult to make evidence-based conclusions about its efficacy to improve dietary intake, eating behavior, and nutritional risk in this specific population. More studies are needed to better understand the importance of nutritional counseling in athletes given the unique risks and consequences associated with imbalanced nutrition and nutrition misinformation affecting eating behaviors. Randomized controlled trials of sufficient size and heterogeneity, including all genders and a variety of sports are needed. As well, future NC interventions should investigate theory-based counseling methods tailored according to factors such as type of sport, level of competition, and age.

## Data availability statement

The original contributions presented in the study are included in the article/supplementary material, further inquiries can be directed to the corresponding author.

## Author contributions

PQ and CF: conceptualization. SF, PQ, EP, LDCLN, CF, and MG: methodology. SF, EP, LDCLN, MG, and CF: investigation. SF, EP, and LDCLN: data curation. SF, MG, and CF: writing – original draft preparation. SF, EP, LDCLN, PQ, MG, CF, and AT: writing – review and editing. CF: supervision. All authors contributed to the article and approved the submitted version.
